# 3′‐Sialyllactose Prevents Atherosclerosis by Attenuating Chronic Inflammation via the Gut‐Immune‐Cardiovascular Axis in *LDLR* ^−/−^ Mice

**DOI:** 10.1002/fsn3.72053

**Published:** 2026-07-01

**Authors:** Yingying Zhuang, Wei Zhang, Linlin Zhou, Hui Shu, Wenqing Bo, Yiding Wang, Xinyuan Huang, Xinning Zhao, Hanying Zheng, Dongbei Guo, Xiaoxuan Chen, Lili Pan, Hongwei Li, Xinyue Wang

**Affiliations:** ^1^ Department of Clinical Nutrition, Zhongshan Hospital (Xiamen) Fudan University Xiamen China; ^2^ Xiamen Clinical Research Center for Cancer Therapy Xiamen China; ^3^ Xiamen Key Laboratory of Biotherapy Xiamen China; ^4^ State Key Laboratory of Vaccines for Infectious Diseases, Xiang An Biomedicine Laboratory, National Innovation Platform for Industry‐Education Integration in Vaccine Research, School of Public Health, Xiamen University Xiamen China

**Keywords:** 3′‐sialyllactose, atherosclerosis, chronic inflammation, gut‐immune‐cardiovascular axis, high‐cholesterol diet, low‐density lipoprotein receptor knockout mice

## Abstract

3′‐sialyllactose (3′‐SL) has demonstrated potential in regulating glycolipid metabolism and improving intestinal immunity, thereby exhibiting anti‐atherosclerotic (AS) properties. However, the mechanism by which 3′‐SL exerts its anti‐AS effects through the regulation of inflammatory signaling via intestinal immunity remains unknown. Herein, 40 male low‐density lipoprotein receptor knockout (*LDLR* ^−/−^) mice were randomized into five groups: normal diet (ND), high‐cholesterol diet (HCD), and three 3′‐SL groups (low dose SLL, medium dose SLM, high dose SLH). After 12 weeks, atherosclerotic plaque formation, serum lipids including total cholesterol (TC), triglycerides (TG), low‐density lipoprotein (LDL), high‐density lipoprotein (HDL), and inflammatory cytokines including interleukin‐6 (IL‐6), interleukin‐1β (IL‐1β), interleukin‐10 (IL‐10) were measured. Multi‐omics analyses (gut microbiota 16S sequencing, colonic transcriptomics, and metabolomics) were performed. Compared with the HCD group, 3′‐SL L intervention dose‐dependently and significantly reduced aortic lipid deposition in *LDLR* ^−/−^ mice, lowered serum TG and LDL levels, increased HDL, while markedly decreasing pro‐inflammatory cytokines IL‐6 and IL‐1β and elevating the anti‐inflammatory cytokine IL‐10. Using the SLM group as representative for mechanistic exploration, it was found that 3′‐SL reshaped the gut microbiota structure, significantly increasing the abundance of beneficial *Akkermansia* and reducing the *Firmicutes/Bacteroidetes* ratio, thereby restoring microbial balance. Integrated multi‐omics analysis further revealed that 3′‐SL drove colonic transcriptomic‐metabolic reprogramming, with enriched key pathways mainly involving immune regulation (e.g., *Ccl2, Il2ra*) and lipid metabolism (e.g., *Kng1, C6*). Differential metabolites showed significant correlations with immune‐ and cardiovascular‐related genes. Among these, *Ccl2* (MCP‐1), as a key molecule linking gut immune signals to coronary artery pathology, was suppressed in expression, which may directly reduce the recruitment of monocytes/macrophages into the subendothelial space, thereby inhibiting the initiation of atherosclerosis. Collectively, 3′‐SL exhibits significant preventive effects against chronic inflammation in *LDLR* ^−/−^ mice with HCD‐induced atherosclerosis. These effects are mediated through synergistic regulation of metabolic, inflammatory, and microbial pathways along the gut microbiota‐immune‐cardiovascular axis. This axis integrates gut microbial metabolism, immune responses, and transcriptional signaling changes, revealing novel targets for the prebiotic 3′‐SL in preventing atherosclerosis. It provides robust preclinical evidence for nutritional intervention strategies targeting the gut microbiota.

## Graphical abstract

3′‐SL intervention in HCD‐fed *LDLR* ^−/−^ mice reduces atherosclerosis, improves metabolic parameters, and suppresses inflammation. Multi‐omics integration shows that 3′‐SL remodels gut microbiota (↓ *Firmicutes*/*Bacteroidota*, ↑ *Akkermansia*), alters colonic metabolites and gene expression (*Kng1*, *Ccl2*, *Il2ra*, *C6*), and coordinates the gut‐immune‐cardiovascular axis to prevent chronic inflammation and atherosclerosis.

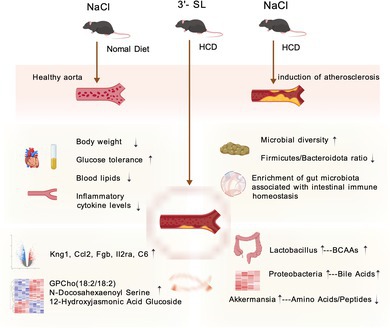

## 1 Introduction

Chronic inflammation has been described as meta‐inflammation (Hotamisligil 2017), believed to originate from evolutionarily conserved nutrient‐sensing and immune signaling pathways. It can lead to tissue damage, organ dysfunction, and the persistence of pathological states (Bhattarai et al. 2024; Itoh et al. 2022). Chronic inflammation serves as a core driver in the development of atherosclerosis (AS), permeating the entire atherosclerotic process. It primarily contributes to atherosclerosis by influencing lipid metabolism and the biological activity of the vascular wall (Yu et al. 2015).

Epidemiological studies reveal that widespread consumption of high‐cholesterol diet (HCD) can induce chronic inflammation, activate immune cells in the small intestine, trigger mucosal immunity, and lead to systemic immunosuppression and inflammatory responses (Furusawa et al. 2013). Related studies have demonstrated that HCD can cause chronic inflammatory diseases such as encephalitis (Kim et al. 2012), pneumonia (Fang et al. 2017), and cardiovascular diseases. Persistent inflammation in blood vessels not only disrupts vascular endothelial homeostasis but also accelerates atherosclerotic plaque progression through mechanisms like monocyte infiltration and foam cell formation (Roy et al. 2022). Hypercholesterolemia is a well‐established risk factor for atherosclerotic cardiovascular disease (Goldstein and Brown 2015). Furthermore, inflammatory markers independently predict cardiovascular disease risk beyond low‐density lipoprotein levels (Ferrucci and Fabbri 2018), suggesting that suppressing inflammatory responses may represent a viable therapeutic approach for AS (Carracedo et al. 2019). Consequently, dietary‐targeted modulation of chronic inflammation has emerged as a critical strategy for AS prevention and management (Libby 2021).

In recent years, the physiological functions of the gut microbiota as the “second genome” have garnered significant attention (Sommer and Bäckhed 2013). The gut microbiota participates in multiple processes within the body, including digestion, absorption, metabolism, and immunity. Healthy gut microbiota regulate host immune homeostasis through metabolic products, maintain intestinal barrier integrity, thereby blocking “gut‐derived inflammation” from damaging the circulatory system (de Vos et al. 2022). Once gut microbiota balance is disrupted, alterations in its structure, function, and metabolic activity can affect metabolism and immunity, triggering various diseases (Chen et al. 2021). Thus, a high‐cholesterol diet can induce gut dysbiosis, intestinal immune dysregulation, and vascular lipid deposition, potentially contributing to AS. Based on this, probiotic intervention to reshape the gut microbiota ecosystem may represent a novel therapeutic target for interrupting this pathological chain reaction.

3′‐sialyllactose (3′‐SL) is one of the oligosaccharides present in high concentrations in human milk. It can reach the colon via the small intestine and is absorbed by the gut microbiota as a prebiotic. 3′‐SL, a predominant sialylated human milk oligosaccharide, has demonstrated potential in mitigating metabolic disorders and intestinal inflammation through its prebiotic properties ^[16]^. However, its role in modulating chronic inflammation within the context of atherosclerosis (AS), particularly through a systematic gut‐immune‐cardiovascular axis, remains largely unexplored. Given that chronic inflammation is a cornerstone of AS pathogenesis and that dietary strategies targeting the gut microbiota hold promise for AS management, we hypothesize that 3′‐SL may modulate systemic inflammation and immune homeostasis by regulating gut microbiota homeostasis and barrier function, thereby controlling chronic inflammation through the “gut‐immune‐cardiovascular axis” to slow AS progression. To investigate the preventive effects on chronic inflammation during AS progression, we selected low‐density lipoprotein receptor knockout (*LDLR* ^−/−^) mice based on a well‐established pathological basis: the low‐density lipoprotein receptor (LDLR) is a core molecule regulating cholesterol homeostasis, maintaining lipid balance (Brown and Goldstein 1986). LDLR deficiency directly causes rapid plasma LDL accumulation, accelerates cholesterol deposition in the endothelium, triggers foam cell formation, and amplifies the HCD‐induced inflammatory cascade (Chandra 2021).

We fed *LDLR* ^−/−^ mice a HCD to model the progression from health to AS, concurrently administering 3′‐SL intervention. This aims to investigate potential mechanisms by which 3′‐SL prevents or mitigates HCD‐induced chronic inflammation in *LDLR* ^*−/−*^ mice. Through metabolomics and transcriptomics technologies, along with analyzing correlations between gut microbiota function and serum inflammatory cytokines/metabolites, we will further investigate its effects on immune‐related signaling pathways. This will provide theoretical support for applying 3′‐SL as a prebiotic to alleviate chronic inflammation in cardiovascular diseases, thereby delaying the onset and progression of AS.

## 2 Materials and Methods

### 2.1 Source of Materials

3′‐SL [specification: ≥ 93% HPLC] were provided by CABIO Biotech (Wuhan) Co. Ltd. According to the manufacturer's certificate of analysis, the endotoxin level of the product was below 10 EU/g (Turck et al. 2020), meeting the safety requirement for oral administration in experimental animals (Kim et al. 2018; Turck et al. 2020).

### 2.2 Ethics Statement

All experimental procedures were carried out in accordance with the guidelines of the Institutional Animal Care and Use Committee of the Laboratory Animal Center of Xiamen University and the International Association of Veterinary Editors guidelines for the Care and Use of Laboratory Animals. Protocols for animal use were reviewed and approved by the Animal Ethical and Welfare Committee of the Laboratory Animal Center of Xiamen University (Approval No. XMULAC20240172).

### 2.3 Animal Experiments

Male “*LDLR* ^−/−^” mice (*n* = 40, 7 weeks old upon arrival) were purchased from Cyagen Biosciences (Suzhou) Inc. and reared at 20°C–24°C, 10%–60% humidity and a 12‐h light–dark cycle. After 1 week of adaptive feeding (at 8 weeks of age), the mean body weight at the start of intervention was 23.65 ± 1.40 g. Eight mice were selected to be fed on a normal diet (ND) (Beijing Keao Xieli Feed Co. Ltd.; Beijing FeedCertificate [2018], 0673) for 12 weeks by random number method after 1 week of adaptive feeding. The remaining 32 mice fed on a high‐cholesterol diet [Synergetic Pharmaceutical & Bioengineering Co. Ltd.], in which the proportion of fat is 20.06% and the proportion of cholesterol is 1.25% were divided into four groups, with 8 mice in each group: the HCD group and three 3′‐SL intervention groups: 3′‐SL low‐dose (SLL), 3′‐SL medium‐dose (SLM) and 3′‐SL high‐dose (SLH) groups.

The three doses of 3′‐SL (low dose: 40.5; medium dose: 81.0; high dose: 162.0 mg/kg/day) were selected based on preliminary experiments and allometric scaling. In our previous pilot study, an effective dose of free sialic acid (SA) was established at 40 mg/kg/day, derived from the effective dose of bird's nest intervention (an SA‐rich natural product) in our laboratory (Zhang et al. 2026). Given that 3′‐SL (molecular weight 633.55) contains one sialic acid moiety per molecule, the equimolar dose of 3′‐SL relative to free SA (molecular weight 309.27) was calculated as 40 mg/kg × (633.55 ÷ 309.27) ≈81.8 mg/kg. Thus, 81.0 mg/kg was chosen as the medium dose. The low and high doses were set as 0.5‐fold (40.5) and 2‐fold (162.0 mg/kg) of the medium dose, respectively, to establish a dose–response relationship. Published toxicological data indicate that 3′‐SL (including its sodium salt form) exhibits a high margin of safety, with a reported No Observed Adverse Effect Level (NOAEL) exceeding 2000 mg/kg BW/day (Kim et al. 2018). All three doses used in the present study are well below this established safety threshold, confirming their suitability for experimental use. For gavage administration, 3′‐SL was freshly dissolved in sterile 0.9% normal saline at the concentrations specified in Table 1. The ND and HCD groups were given 0.9% saline by gavage at a volume of 0.1 mL/10 g mouse body weight. All treatments were administered once daily for 12 weeks. Weight was measured weekly, and an oral glucose tolerance tests (OGTT) were conducted at weeks 4, 8, and 12. The mice were administered a 20% glucose via gavage according to their body weight (10 μL/g) after fasting for 12 h. Blood glucose concentrations were then measured by taking blood samples from the tail veins of mice at 0, 30, 60, and 120 min after glucose administration using a blood glucose meter (Sanuo Biological Sensing Co. Ltd). The area under the curve (AUC) was calculated for each OGTT.

**TABLE 1:** Animal experimental protocol.

Group	*N*	Test substance	Supplementation dose in mice (mg/day kg)	Gavage concentration (mg/mL)
Normal diet (ND)	8	Normal saline	—	—
High‐cholesterol diet group, (HCD)	8	Normal saline	—	—
3′‐SL low‐dose (SLL)	8	3′‐Sialyllactose	40.50	4.05
3′‐SL medium‐dose (SLM)	8	3′‐Sialyllactose	81.00^a^	8.10
3′‐SL high‐dose (SLH)	8	3′‐Sialyllactose	162.00	16.200

^a^ The three doses were selected based on equimolar conversion from an effective dose of free sialic acid (40 mg/kg/day) and allometric scaling. The medium dose (81.0 mg/kg/day) corresponds to the equimolar dose of 40 mg/kg free sialic acid. The low and high doses were set as 0.5‐fold and 2‐fold of the medium dose, respectively. Detailed calculation and justification are provided in Section 2.3 (Animal experiments).

### 2.4 Sample Collectiong and Index Testing

When the intervention ended, the mice fasted for 12 h, were anesthetized with 4% isoflurane (Cat# R510‐22, Shenzhen Reward Life Technology Co. Ltd) and then euthanized through rapid spinal dislocation. The eyeballs were removed to collect blood. Organs (liver, spleen, kidney, colon, and so on) were collected, rinsed with 0.9% saline, and weighed rapidly. The collected blood was centrifuged at 2000 r/min at 4°C for 15 min, and the resultant supernatant was collected to measure biochemical indicators. All samples were frozen at −80°C for later use.

### 2.5 Serum Biochemical Indicators

The contents of total cholesterol (TC), triglycerides (TG), low‐density lipoproteins (LDL), and high‐density lipoproteins (HDL) in serum were detected by an automatic biochemical analyzer (Mindray BS‐220, Shenzhen Mindray Bio‐Medical Electronics Co. Ltd., China) with dedicated kits (TC kit, Cat# BTK063; TG kit, Cat# BTK009; LDL kit, Cat# BTK012; HDL kit, Cat# BTK011; *Bioswamp*, Wuhan, China). The interleukin‐1β (IL‐1β), interleukin‐6 (IL‐6), interleukin‐10 (IL‐10), and tumor necrosis factor‐alpha (TNF‐α) in serum were measured using mouse enzyme‐linked immunosorbent assay kits (IL‐1β kit, Cat# MU30369; IL‐6 kit, Cat# MU30009; IL‐10 kit, Cat# MU30055; TNF‐α kit, Cat# MU30030; *Bioswamp*, Wuhan, China), according to the manufacturer's instructions.

### 2.6 Oil Red O Staining (Oil‐Red) of the Total Length of the Aorta

After euthanasia, the aorta was carefully dissected from the ascending aorta to the iliac bifurcation under a stereomicroscope. Periadventitial adipose tissue was removed as much as possible using forceps (Cat# QX1020, Servicebio) and dissecting scissors (Cat# QX1424, Servicebio). The aorta was fixed in 4% paraformaldehyde fixative (Cat# G1101, Servicebio) for at least 24 h at room temperature. After fixation, the samples were washed twice with phosphate‐buffered saline (PBS). The aorta was then opened longitudinally along the vessel wall with dissecting scissors. The opened aortas were briefly rinsed with tap water for 5 s, immersed in 60% isopropyl alcohol (SCRC, 80109218) for 3 s, and then stained with Oil red O staining solution (Servicebio, G1015) at 37°C in the dark for 60 min. After staining, the aortas were transferred into 60% isopropyl alcohol for differentiation. Differentiation was performed for approximately 1 min until the lipid plaques in the vessel lumen appeared orange‐red or bright red while the background became nearly colorless. The differentiation was terminated by washing with distilled water. The stained aortas were placed on a glass slide on a black or white background plate with a scale ruler, spread flat, and photographed using a digital camera (Canon D70) under consistent lighting conditions. The scale ruler was included in each photograph for calibration.

### 2.7 16S rDNA Sequencing

Colon contents were swiftly frozen in liquid nitrogen postsampling and stored at −80°C. DNA was extracted from these samples using the HiPure Stool DNA kit (Cat#: D314102, Magen, Guangzhou, China). The conserved 16S rDNA gene region (V3: 341F, CCTACGGGNGGCWGCAG; V4: 806F, GGACTACHVGGGTATCTAAT) was polymerase chain reaction (PCR)‐amplified using suitable primers with barcodes. The resulting amplicons were purified, quantified, and subjected to equimolar pooling for paired‐end sequencing (PE250) on an Illumina sequencer (Life Technologies, CA, USA). Raw reads were filtered, assembled, and processed using FASTP (v. 0.18.0). Clean tags were clustered into operational taxonomic units (OTUs) with a ≥ 97% similarity threshold using the UPARSE pipeline (v. 9.2.64). Chimeric tags were removed using the UCHIME algorithm, and the remaining tags underwent downstream analyses. The most abundant sequence in each OTU served as a representative sequence. Following OTU determination, the gut microbiota indices (community composition, alpha diversity, beta diversity, indicator species, and intestinal flora function) were assessed using the Omicsmart platform (Majorbio Bio‐pharm Technology Co. Ltd. Shanghai, China; https://www.majorbio.com).

### 2.8 Transcriptomic Analysis of Colon

Total RNA was extracted using the TRIzol reagent kit (Cat#: 15596026, Invitrogen, Carlsbad, CA, USA) following the manufacturer's protocol. The RNA quality was assessed using an Agilent 2100 bioanalyzer (Agilent Technologies, Palo Alto, CA, USA) and verified by RNase‐free agarose gel electrophoresis. After total RNA extraction, eukaryotic mRNA was enriched using oligo(dT) beads. The enriched mRNA was fragmented into short fragments using fragmentation buffer and reverse‐transcribed into cDNA using the NEB Next Ultra RNA Library Prep Kit for Illumina (NEB #7530, New England Biolabs, Ipswich, MA, USA). The purified double‐stranded cDNA fragments were subjected to end repair, A tailing, and ligation with Illumina sequencing adapters. The ligated sequences were purified using AMPure XP beads (1.0×) and subsequently amplified by PCR. The resulting cDNA library was sequenced by Majorbio Bio‐pharm Technology Co. Ltd. (Shanghai, China) using an Illumina Novaseq6000 sequencer.

### 2.9 Metabolomics Analysis of the Colon

Mouse tissue samples were preprocessed according to a previously described method. Twenty‐six ultra‐high‐performance liquid chromatography–tandem mass spectrometry (UHPLC–MS/MS) analyses were performed by Majorbio Bio‐pharm Technology Co. Ltd. (Shanghai, China) using a Vanquish UHPLC system (Thermosphere, Germany) coupled with an Orbitrap Q ExactiveTM HF‐X mass spectrometer (Thermo Fisher, Germany). The raw data files generated using UHPLC–MS/MS were processed using Compound Discoverer 3.1 (CD 3.1; Thermo Fisher Scientific) to perform peak alignment, peak picking, and quantitation for each metabolite. Normalized data were used to predict the molecular formulae based on additive ions, molecular ion peaks, and fragment ions. To obtain accurate qualitative and relative quantitative results, the detected peaks were matched to the spectra in the mzCloud (https://www.mzcloud.org/), mz Vault, and MassList databases.

### 2.10 Statistical Analysis

Data were analyzed using SPSS 22.0 and R statistical software. Repeated measures data were analyzed for variance using multivariate analysis. For normally distributed data with homogeneous variance, a one‐way analysis of variance (oneway ANOVA) was used to compare differences between groups, and Fisher's least significant difference method (LSD) was used for pairwise comparisons between groups. In the case of normally distributed data for which the variance was nonhomogeneous, we used Dunnett's T3 test for pairwise comparisons between the groups. For non‐normally distributed data, we used the non‐parametric Kruskal–Wallis *H*‐test for comparison between groups, and the Nemenyi method was used for pairwise comparison of the overall means between groups. Differences were considered significant at *α* = 0.05 level.

## 3 Result

### 3.1 Weight, Oral Glucose Tolerance Test (OGTT) and Blood Lipids

**FIGURE 1:**
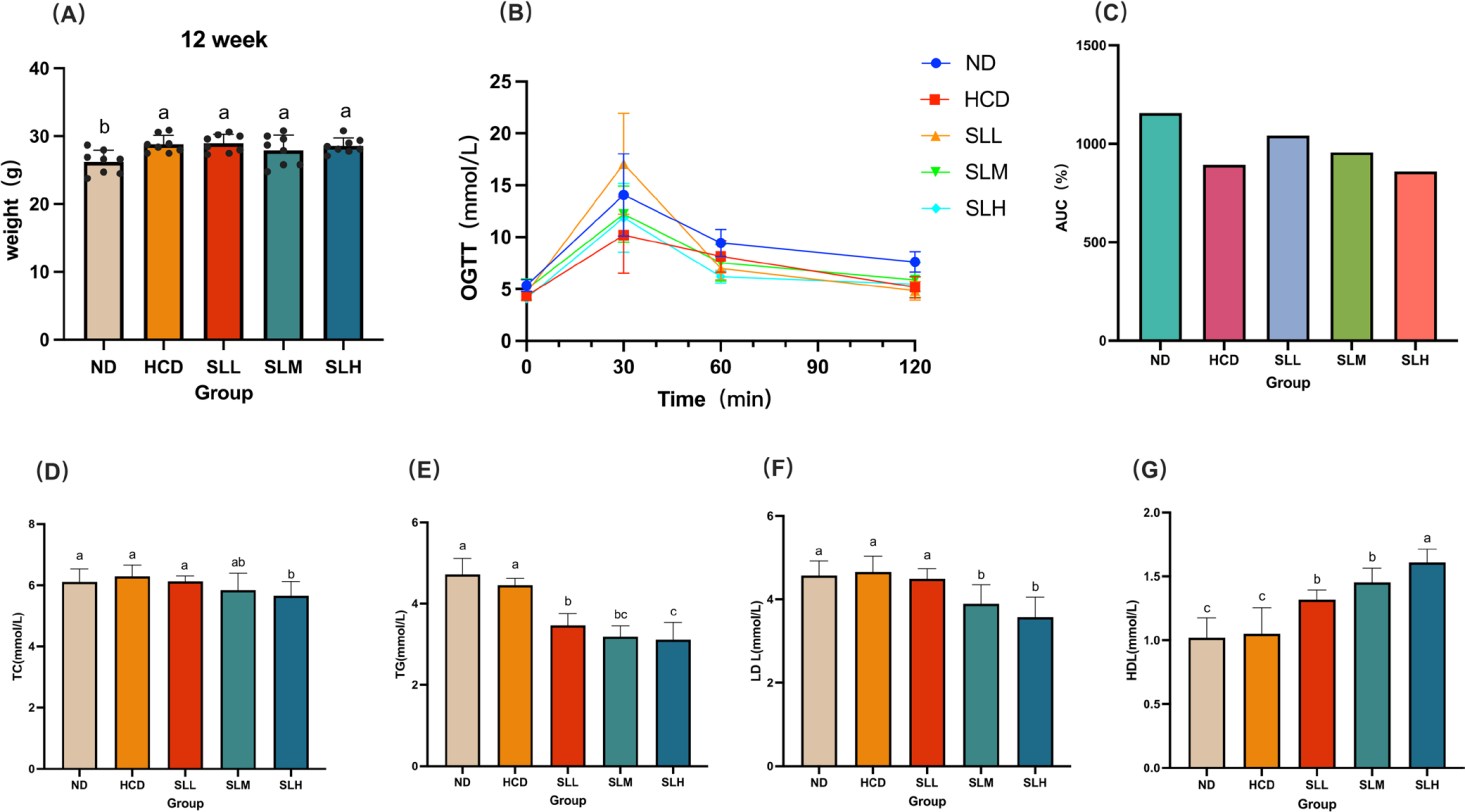
Effect of 3′‐SL intervention on body weight, blood glucose levels and blood lipid in *LDLR* ^*−/−*^ mice fed a high‐cholesterol diet. (A) Body weight in each group after 12 weeks of intervention. (B) OGTT (oral glucose tolerance tests). (C) The relative value of the area under the OGTT curve (with the HCD group as the reference). (D) TC (total cholesterol). (E) TG (triglycerides). (F) LDL (low‐density lipoprotein). (G) HDL (high‐density lipoprotein). The difference between values with completely different superscripts was statistically significant, *p* < 0.05. Data are means ± SD (*n* = 8).

As shown in Figure 1A–C, after 12 weeks of intervention, the HCD group exhibited significantly higher body weight compared to the ND group (*p* < 0.05). Body weights in all intervention groups were similar to that of the HCD group, with the SLM group showing slightly lower weight than the HCD group, though this difference was not statistically significant (*p* > 0.05). Changes in OGTT curves and comparisons of area under the curve (AUC) indicated that, compared with the HCD group, the intervention groups demonstrated superior glucose tolerance, exhibiting a dose–response relationship.

As shown in Figure 1D–G, compared with the HCD group, the SLH group exhibited a significant reduction in TC levels (*p* < 0.05), while the reductions in the SLL and SLM groups were not statistically significant (*p* > 0.05). TG levels in all intervention groups showed a marked decrease relative to the HCD group (*p* < 0.05). LDL levels in all intervention groups decreased with increasing dosage, exhibiting a clear dose–response relationship, with statistically significant reductions observed in the SLM and SLH groups (*p* < 0.05). HDL levels in the intervention groups showed a marked increase (*p* < 0.05). These findings indicate that 3′‐SL intervention reduces circulating TG and LDL while elevating HDL, playing a crucial role in lipid metabolism.

### 3.2 Inflammatory Cytokines and Oil Red O Staining (Oil‐Red) of the Aorta

**FIGURE 2:**
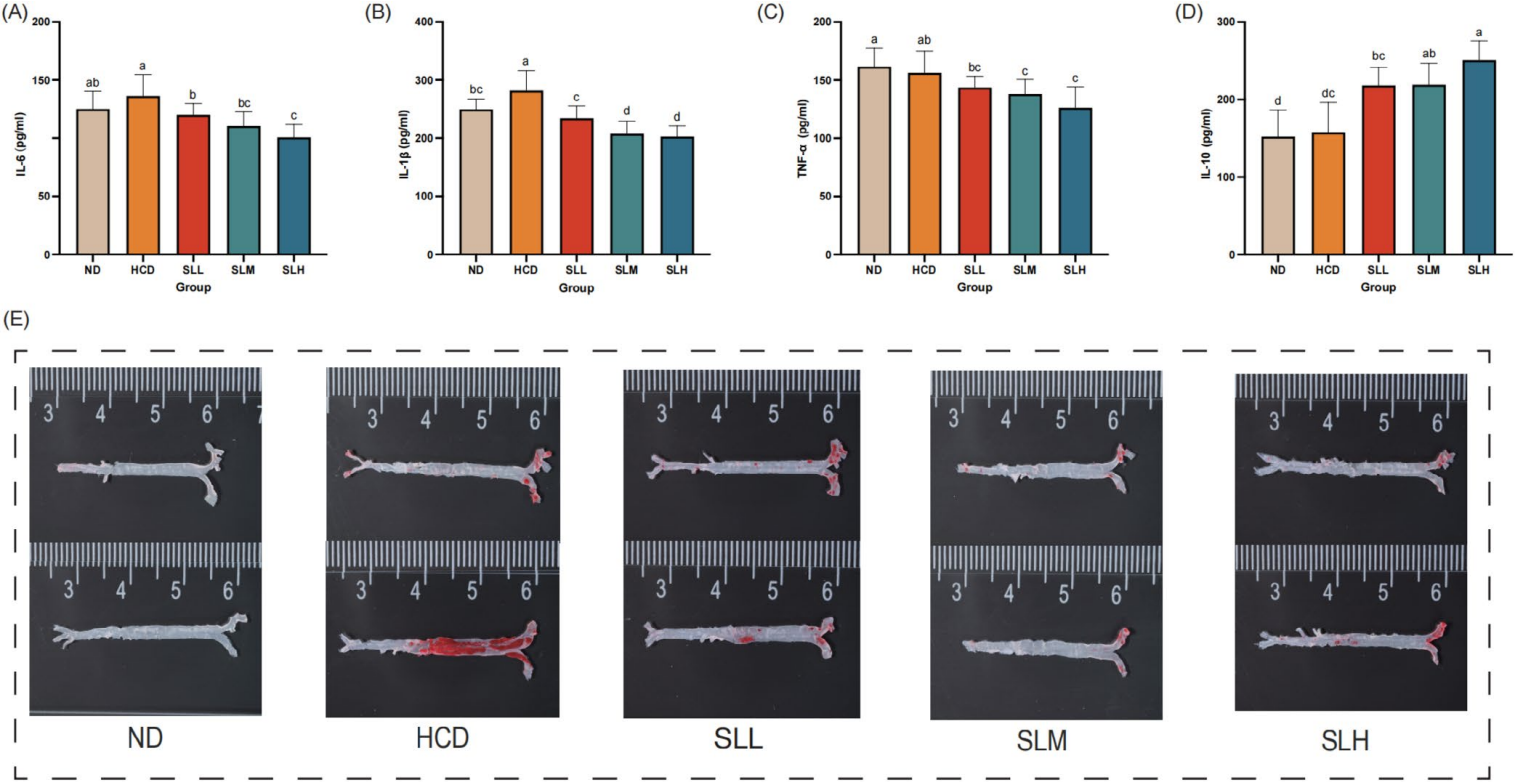
Effects of 3′‐SL intervention on serum inflammatory cytokines and aortic deposits in *LDLR* ^−/−^ mice fed a high‐cholesterol diet. (A) IL‐6; (B) IL‐1β; (C) tumor necrosis factor‐α (TNF‐α); (D) IL‐10; (E) the aorta of ND group, HCD group, SLL group, SLM group, and SLH group. The difference between values with completely different superscripts was statistically significant, *p* < 0.05. Data are means ± SD (*n* = 8).

As shown in Figure 2A–D, inflammatory cytokines were elevated in the HCD group. Compared with the HCD group, the levels of IL‐6 and IL‐1β were significantly reduced in the intervention groups (*p* < 0.05). The levels of TNF‐α differed significantly between the SLM, SLH, and HCD groups, with statistically significant differences (*p* < 0.05). Compared to the HCD group, the level of the anti‐inflammatory factor IL‐10 was significantly elevated. This indicates that in mice fed a high‐fat, high‐cholesterol diet, 3′‐SL intervention reduced pro‐inflammatory cytokines and increased anti‐inflammatory cytokines.

The moderate‐dose group was selected as representative for further mechanism exploration. Oil‐red O staining was used to assess the severity of aortic lesions throughout the entire aorta, evaluating the pathological status of AS plaques at different local sites within the vessel. As shown in Figure 2E–G, significant fatty deposition was observed in the aorta of the HCD group. Compared to the HCD group, mice in the SLM group exhibited reduced areas of Oil‐red O staining and less intense staining in the aorta, suggesting a decrease in lipid deposition following intervention.

Because the SLM and SLH groups showed comparable efficacy with no significant differences in the key phenotypic parameters (aortic plaque area, serum lipids, and inflammatory cytokines), the SLM group was selected as the representative dose for subsequent multi‐omics mechanistic exploration (gut microbiota, colonic transcriptomics, and metabolomics).

### 3.3 Gut Microbiota

**FIGURE 3:**
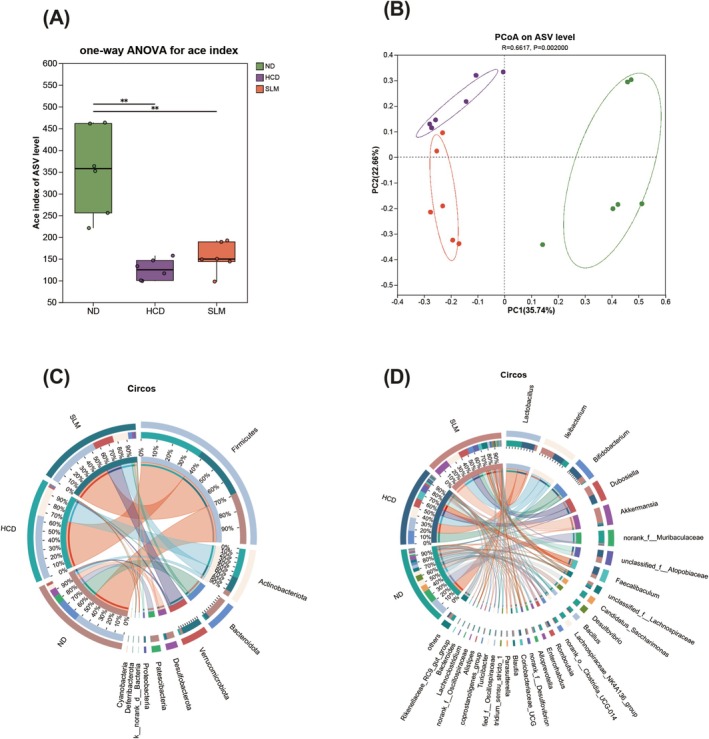
Effects of 3′‐SL intervention on gut microbiota in *LDLR* ^*−/−*^ mice fed a high‐cholesterol diet (*n* = 6). (A) alpha diversity; (B) beta diversity; (C) relative abundance bar plot at the phylum level; (D) relative abundance at the genus level.

The α‐diversity index reflects the richness and evenness of microbial communities, while β‐diversity analysis enables comparative analysis of GM community composition across different samples. Principal Coordinates Analysis (PCoA) is one of the primary methods for β‐diversity analysis. As shown in Figure 3A,B, alpha and beta diversity analyses revealed that species richness in the HCD and SLM groups was significantly lower than in the ND group. The HCD and SLM groups exhibited relatively minor differences in community structure, with SLM showing higher species richness than HCD, though this difference was not statistically significant (*p* > 0.05).

As shown in Figure 3C,D, species analysis revealed a significant increase in *Firmicutes* at the phylum level in the HCD group, with other bacterial groups becoming marginalized, suggesting gut microbiota imbalance in the HCD group. Compared to the HCD group, the SLM group showed significantly increased abundance of *Verrucomicrobiota* and *Bacteroidetes* phyla, while *Firmicutes* and *Actinobacteria* phyla decreased. The *Firmicutes*/*Bacteroidetes* ratio was reduced in the SLM group. At the genus level, compared to the HCD group, the SLM group showed increased abundance of genera such as *Akkermansia*, while genera like *Bifidobacterium* and *Lactobacillus* decreased.

**FIGURE 4:**
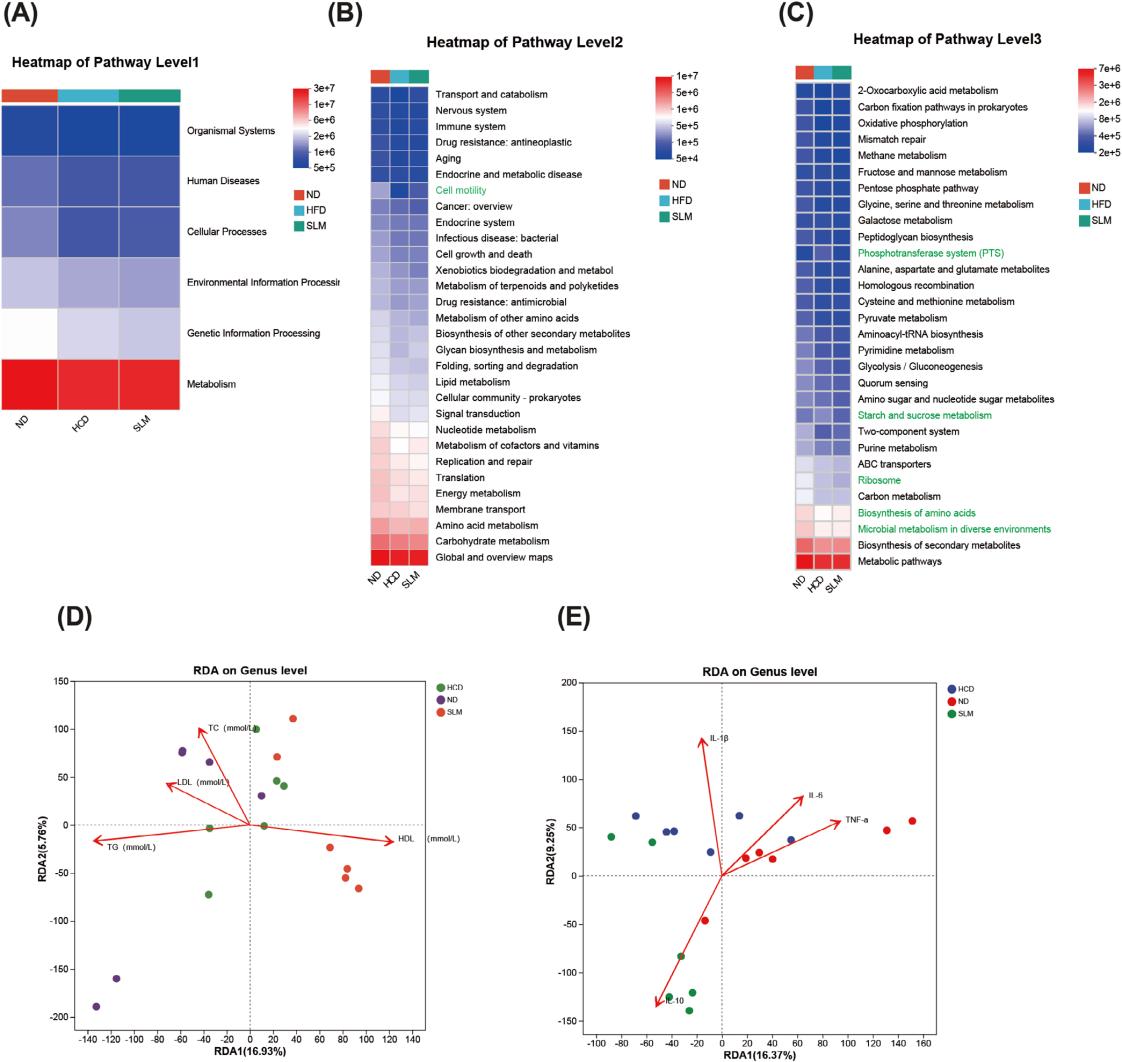
Functional prediction of colonic microbiota based on PICRUSt2 reference sequences. (A) Level 1 functional classification of colonic microbiota; (B) level 2 functional classification of colonic microbiota; (C) level 3 functional classification of colonic microbiota; (D) canonical correspondence analysis of correlations between serum TC, TG, LDL, and HDL with intestinal flora; (E) canonical correspondence analysis of correlations between serum IL‐1β, IL‐6, IL‐10, and TNF‐α with intestinal flora.

As shown in Figure 4A–C, compared to the HCD group, at Level 1, the SLM diet did not show significant differences in HCD‐induced gut microbiota functional pathways. At Level 2, the SLM group showed increased abundance only in the Cell motility pathway compared to the HCD group. However, at Level 3, the SLM group exhibited increased enrichment in gut microbiota functional pathways supporting intestinal energy metabolism and enhancing immune homeostasis, including Microbial metabolism in diverse environments, Biosynthesis of amino acids, Ribosome, Starch and sucrose metabolism, and Phosphotransferase system (PTS).

As shown in Figure 4D,E, compared with the ND group, the HCD group showed a significant positive correlation between gut microbiota and elevated lipid levels (TG [Envfit *r* ^2^ = 0.4677, FDR‐corrected *p* = 0.014], TC [Envfit *r* ^2^ = 0.2769, FDR‐corrected *p* = 0.096]) and a significant negative correlation with HDL (Envfit *r* ^2^ = 0.4050, FDR‐corrected *p* = 0.027) (LDL showed a consistent trend but did not reach statistical significance, Envfit *r* ^2^ = 0.1644, FDR‐corrected *p* = 0.289). Following SLM intervention, the gut microbial structure shifted toward that of the ND group, and the correlations with lipid indices were attenuated, suggesting that 3′‐SL can ameliorate lipid‐metabolism disorders via microbiota modulation.

Relative to the ND group, the HCD group's microbiota was significantly associated with increased serum inflammatory cytokines (IL‐1β [Envfit *r* ^2^ = 0.4361, FDR‐corrected *p* = 0.016], IL‐10 [Envfit *r* ^2^ = 0.3419, FDR‐corrected *p* = 0.046]) (IL‐6 and TNF‐α showed similar trends but were not statistically significant, Envfit *r* ^2^ = 0.1674/0.2074, FDR‐corrected *p* = 0.263/0.196).

SLM intervention markedly reshaped microbial composition. Specifically, the enrichment of beneficial genera such as Akkermansiaand the restoration of Bacteroidetes abundance were associated with a weakened positive correlation between the microbial community and pro‐inflammatory IL‐1β, and a strengthened negative correlation with anti‐inflammatory IL‐10. This indicates that SLM suppresses chronic inflammation through the microbiota‐immune axis, with *Akkermansia* potentially playing a pivotal role.

### 3.4 Integrated Analysis of Colon Transcriptome and Metabolome

**FIGURE 5:**
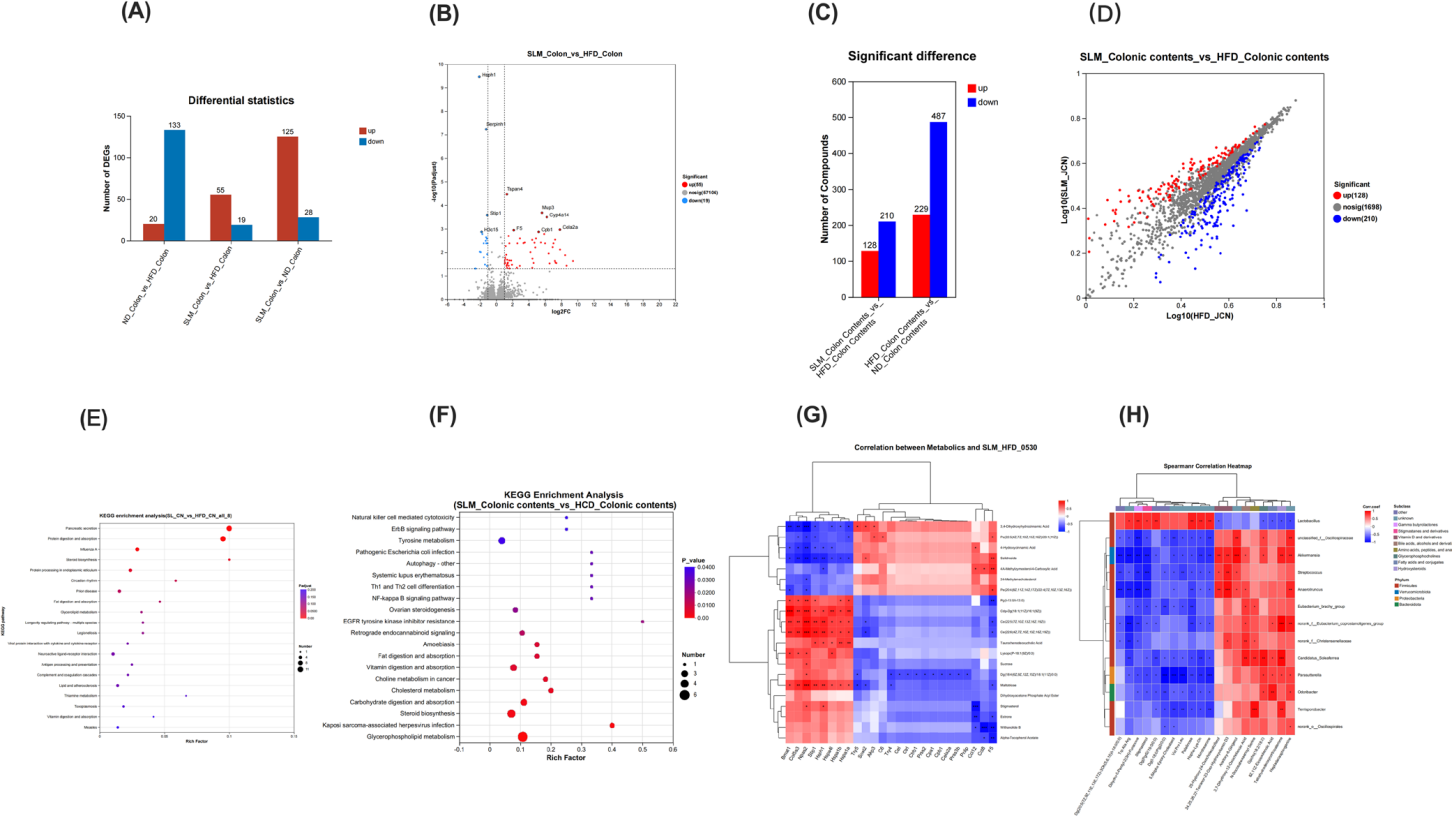
Effects of 3′‐SL intervention on colonic transcription and metabolism in *LDLR* ^−/−^ mice fed a high‐cholesterol diet (*n* = 6). (A, B) Differentially expressed genes statistics: SLM versus HCD; (C, D) differential metabolite statistics: SLM versus HCD; (E) functional analysis of differentially expressed transcripts in colon tissue, top 15 KEGG pathways (SLM group); (F) top 15 enriched KEGG pathways of differentially expressed genes (SLM group); (G) integrated analysis of differentially expressed genes and differential metabolites in pathway enrichment; (H) correlation analysis of the top 20 enriched differential gut microbiota and differential metabolites. Significance levels: *p < 0.05, **p < 0.01, *p < 0.001.

As shown in Figure 5A–D, transcriptomic analysis showed that compared to the HFD group, the ND group exhibited 20 upregulated and 133 downregulated genes, while the SLM intervention resulted in 55 upregulated and 19 downregulated genes relative to HFD. Metabolomic profiling demonstrated that SLM treatment induced 128 upregulated and 210 downregulated metabolites compared to HFD, whereas HFD feeding caused 229 upregulated and 487 downregulated metabolites relative to ND controls.

As shown in Figure 5E,F, differential metabolite pathway enrichment analysis revealed that compared to HCD, the top 15 Kyoto Encyclopedia of Genes and Genomes (KEGG) pathways enriched by differential metabolites were primarily associated with immune regulation, inflammatory response, energy metabolism, lipid metabolism, and hormone synthesis. These pathways improved physiological issues such as inflammatory states, metabolic disorders, and immune dysfunction. The top 15 KEGG pathways enriched for differential genes primarily involved digestive function, immune response, steroid synthesis, circadian rhythm regulation, and neural signaling. These pathways improved metabolic disorders, dysregulated inflammatory responses, and immune system abnormalities induced by high‐cholesterol diet.

### 3.5 Integrated Analysis of Transcriptomics‐Metabolomics and Gut Microbiota‐ Metabolomics

As shown in Figure 5G, joint analysis of enriched differential metabolites and differential genes revealed that multiple metabolites (N‐Docosahexaenoyl Serine, GPCho [18:2/18:2], 12‐Hydroxyjasmonic Acid Glucoside) showed significant correlations with genes related to immune regulation and cardiovascular disease (e.g., *Kng1*, *Ccl2*, *Fgb*, *Il2ra*, *C6*). Notably, lipid metabolites and cholesterol derivatives exhibited high correlations with inflammatory cytokines, suggesting these metabolites may regulate systemic inflammation levels by influencing immune gene expression.

As shown in Figure 5H, *Firmicutes* is the dominant gut microbiota, positively correlated with vitamin D and its derivatives, fatty acylglycosides, fatty acids, and their complexes. Among these, *Lactobacillus* exhibits positive correlations with multiple branched‐chain amino acids. *Proteobacteria* and *Bacteroidetes* phyla show positive correlations with carbohydrates and their conjugates, while exhibiting negative correlations with bile acids and their derivatives. *Akkermansia* shows positive correlations with amino acids, peptides, and their analogues, and terpenoid compounds.

## 4 Discussion

Chronic inflammation is a core driver of the initiation and progression of atherosclerosis (AS). This study utilized *LDLR* ^−/−^ mice as experimental animals to investigate the preventive effect of 3′‐SL intervention on inflammation during AS progression. The results demonstrated that compared to the HCD group, 3′‐SL treatment significantly reduced aortic lipid deposition in *LDLR* ^−/−^ mice, an effect likely attributable to its effective control of chronic inflammation. The following discussion will explore how 3′‐SL prevents the HCD‐induced chronic inflammatory state in *LDLR* ^−/−^ mice through the “gut‐immune‐cardiovascular axis,” aiming to provide insights into the application of 3′‐SL for AS prevention and broader chronic disease management.

### 4.1 3′‐SL Restores Gut Microbiota Balance to Alleviate Systemic Inflammatory Conditions

The gut microbiota significantly influences the development and function of the immune system (Zeng et al. 2023). HCD‐induced gut dysbiosis, alterations in microbial structure, and increased intestinal permeability are key mechanisms triggering systemic inflammatory responses (Li et al. 2021). Gut dysbiosis and local inflammation activate immune cells within the intestinal lamina propria, driving them to express specific chemokine receptors and migrate to distant tissues and organs, thereby inducing systemic inflammation.

This study reveals that 3′‐SL acts on the colonic microbiota, significantly enriching beneficial bacteria such as *Akkermansia* and *Bacteroidetes*, while suppressing certain pro‐inflammatory species within *Firmicutes*. This markedly corrected the dysregulated *Firmicutes/Bacteroidetes* (F/B) ratio and countered dysbiosis. Given that decreased *Bacteroidetes* and increased *Firmicutes* are associated with AS development (Yang et al. 2015; Crovesy et al. 2020; Magne et al. 2020), this optimized microbial structure is directly linked to AS pathogenesis. *Lactobacillus* promotes the production of branched‐chain amino acid metabolites, contributing to the regulation of intestinal immune homeostasis. Simultaneously, *Akkermansia*, acting as an “intestinal guardian,” enhances the secretion of short‐chain fatty acids (SCFAs) to modulate inflammatory responses, thereby influencing the progression of obesity, diabetes, and cardiovascular metabolic diseases (He et al. 2023). *Akkermansia* also releases outer membrane vesicles (OMVs) into intestinal epithelial cells, stimulating the expression of tight junction proteins and mucins (Wang et al. 2023). By adhering to the intestinal mucus layer and enhancing the expression of tight junction proteins (e.g., Occludin, Claudin‐1), it effectively reduces endotoxin translocation, thereby suppressing systemic inflammation (Shen et al. 2009). This aligns with our observed decrease in serum pro‐inflammatory cytokines (IL‐6, IL‐1β) and increase in the anti‐inflammatory factor IL‐10 (as shown in Figure 2). IL‐6 and IL‐1β are associated with an elevated risk of cardiovascular events (Ridker et al. 2017) and serve as inflammatory markers for cardiovascular disease. The repair of the intestinal barrier and reduction of inflammation collectively form a first line of defense against AS, preliminarily revealing the mechanism by which 3′‐SL suppresses the initiation of AS inflammation via the “microbiota‐immune” axis.

### 4.2 Multi‐Omics Analysis Reveals the Core Mechanism of 3′‐SL Action via the Gut‐Immune‐Cardiovascular Axis

Integrated multi‐omics analysis in this study outlines a coherent mechanism for the anti‐atherosclerotic effects of 3′‐SL. This mechanism originates from the remodeling of the gut microbiota and ultimately translates into direct improvements in the cardiovascular system.

Our integrated microbiome‐metabolome analysis revealed significant positive correlations between specific *Firmicutes* species and vitamin D (VD) derivatives, fatty acid complexes, and carbohydrate conjugates. These findings suggest that the gut microbiota may exert cardiovascular protective effects by modulating VD metabolism and fatty acid synthesis, thereby influencing immune responses and lipid metabolism. Notably, metabolites like 24,25,26,27‐Tetranor‐23‐Oxo‐Hydroxyvitamin D3 may promote regulatory T cell (Treg) differentiation and function through the VD receptor (VDR) signaling pathway, simultaneously enhancing IL‐10 secretion while suppressing Th1 and Th17 inflammatory responses (Boulkrane et al. 2020). VD also inhibits dendritic cell (DC) maturation, reducing their antigen‐presenting capacity and thereby mitigating excessive immune activation. Indigestible carbohydrate conjugates serve as primary precursors for SCFAs (Topping and Clifton 2001). SCFAs inhibit the nuclear factor kappa‐B (NF‐κB) pathway in macrophages and intestinal epithelial cells via G protein‐coupled receptor 43 (GPR43)/GPR41 receptors (Sung et al. 2017; Thio et al. 2018), reducing the secretion of pro‐inflammatory cytokines such as IL‐6. Amino acids, peptides, and their analogues showed positive correlations with *Akkermansia*. *Akkermansia* can secrete mucinases to break down mucins, releasing amino acids and carbohydrates (Sung et al. 2017). Glutamine acts as a key energy substrate for intestinal epithelial cells, promoting cell proliferation/repair and maintaining the mucus barrier while reducing pathogen penetration. Meanwhile, arginine improves intestinal blood flow via the nitric oxide pathway, synergistically enhancing barrier function (Armstrong et al. 2018). Furthermore, peptide metabolites can directly inhibit the growth of harmful bacteria, maintain microbial balance and reducing inflammatory stimulation.

Most importantly, this study identified key molecular bridges connecting gut‐derived immune signals to coronary artery and cardiovascular pathologies. Joint transcriptomic and metabolomic analysis revealed that differential metabolites were significantly correlated with immune‐related genes (e.g., *Ccl2, Il2ra*) and cardiovascular‐related genes (e.g., *Kng1, C6*). Among these, the chemokine *Ccl2* (Monocyte Chemoattractant Protein‐1, MCP‐1) represents a critical link between systemic inflammation and coronary artery disease (Georgakis et al. 2019). Anti‐inflammatory metabolites originating from the gut and entering the circulation may systemically suppress the expression of *Ccl2* in the vascular wall cells of the aorta and coronary arteries, thereby directly reducing the recruitment and infiltration of monocytes/macrophages into the subendothelial space a key initial step in atherosclerotic plaque formation. Concurrently, changes in the expression of the *Kng1* gene suggest that 3′‐SL might regulate vascular tone, improve endothelial function, and inhibit vascular smooth muscle cell proliferation via the kinin system (Hu et al. 2018). Additionally, the association with the complement component C6 suggests that the intervention may mitigate vascular endothelial damage mediated by the membrane attack complex of the complement system, thereby participating in vascular inflammatory responses and immune regulation (Pilzer et al. 2005; Xiong et al. 2003).

In summary, 3′‐SL not only suppresses systemic inflammation by reshaping the gut microbiota structure but also, through the regulation of the intestinal metabolite profile and local immune gene expression, directly impacts vascular inflammatory cell recruitment, vascular function, and vascular integrity. This synergistic “microbiota‐metabolite‐transcriptome” regulation constitutes the core mechanism by which 3′‐SL exerts its anti‐atherosclerotic effects through the “gut‐immune‐cardiovascular axis.”

### 4.3 Considerations and Limitations for 3′‐SL Use in Patients With Inflammatory Bowel Disease

While the present study demonstrates the anti‐inflammatory and gut microbiota‐modulating effects of 3′‐SL in high‐cholesterol diet‐induced atherosclerosis in *LDLR* ^−/−^ mice, several important considerations must be addressed before extrapolating these findings to patients with inflammatory bowel disease (IBD), including Crohn's disease and ulcerative colitis.

First, the existing evidence on 3′‐SL in the context of IBD remains preliminary. A recent in vitro study using fecal microbiota from pediatric Crohn's disease patients demonstrated that 3′‐SL exhibited a bifidogenic effect and increased the abundance of *Faecalibacterium prausnitzii* and *Blautia* species, which possess anti‐inflammatory properties (Otaru et al. 2024). Additionally, 3′‐SL has been shown to synergistically alleviate intestinal inflammation and barrier dysfunction in ulcerative colitis models by promoting short‐chain fatty acid production through cross‐feeding mechanisms among gut microbiota (Parente et al. 2003; Yang et al. 2025). These findings suggest potential benefits; however, no clinical trials have directly evaluated the efficacy or safety of 3′‐SL supplementation in IBD patients.

Second, the safety profile of 3′‐SL in the context of a disrupted intestinal barrier remains to be fully established. Although 3′‐SL is generally recognized as safe (GRAS) by the U.S. FDA and has been approved as a novel food ingredient by the European Union for the general population (Turck et al. 2020), and although a clinical study in dyspeptic patients with *H. pylori* infection reported that 3′‐SL (up to 20 g/day for 4 weeks) was safe and well tolerated without serious adverse events (Parente et al. 2003), IBD patients may respond differently due to altered gut permeability, dysbiosis, and ongoing mucosal inflammation. In the setting of a compromised intestinal barrier, the theoretical risk of increased bacterial fermentation products, gas production, or potential immune stimulation cannot be entirely excluded. Additionally, individual responses to prebiotics vary substantially depending on baseline gut microbiota composition, and IBD patients exhibit considerable heterogeneity in their microbial profiles (Otaru et al. 2024).

Third, there is currently no established dosing regimen for 3′‐SL in IBD. The doses used in our study (40.5–162.0 mg/kg/day in mice, approximately 6.5–26.0 g/day for a 60 kg human based on allometric scaling) were derived from prebiotic efficacy studies in metabolic disease models. Whether these doses are appropriate, tolerable, or safe in IBD patients with active inflammation remains unknown.

Therefore, while 3′‐SL holds promise as a prebiotic agent for modulating gut microbiota and systemic inflammation, caution is warranted when considering its use in patients with IBD. Future studies should include well‐designed clinical trials specifically in IBD populations, with careful monitoring of gastrointestinal symptoms, inflammatory markers, and gut microbiota dynamics, before any clinical recommendations can be made. Moreover, the timing of supplementation (active disease vs. remission) and potential interactions with concomitant medications (e.g., immunosuppressants, biologics) warrant systematic investigation.

### 4.4 Limitation

This study has several limitations. First, the exclusive use of male mice precludes the evaluation of potential sex differences in the response to 3′‐SL via the gut‐immune‐cardiovascular axis. Female mice undergo regular estrous cycles, during which fluctuations in estrogen and progesterone significantly influence lipid metabolism, inflammatory responses, and gut microbiota composition. These hormonal variations may lead to sex‐dependent differences in the anti‐atherosclerotic effects of 3′‐SL. Therefore, whether the mechanistic insights observed in male mice fully apply to females remains unknown, and future studies should include female *LDLR* ^−/−^ mice to address this gap. Second, while multi‐omics correlations were identified, the causal relationships between the reshaped gut microbiota, the altered metabolites, and the observed cardioprotective effects remain to be established through further mechanistic studies, such as fecal microbiota transplantation experiments. Finally, for clinical translation, the long‐term safety and efficacy of 3′‐SL in humans need to be validated in large‐scale clinical trials.

## 5 Conclusion

This multi‐omics integrated study confirms that 3′‐SL modulates colonic metabolism and transcriptional signaling by reshaping gut microbiota structure and function. Through a cascade regulation mechanism involving the microbiota‐immune‐cardiovascular axis, it blocks HCD‐induced chronic inflammation and AS progression. This discovery not only elucidates the potential cardiovascular protective mechanism of 3′‐SL as a prebiotic but also provides theoretical support and application directions for “gut microbiota‐targeted nutritional interventions” in preventing and treating AS. In summary, our study demonstrates that the prebiotic 3′‐SL represents a promising nutritional strategy for preventing atherosclerosis by regulating the gut‐immune‐cardiovascular axis.

## Author Contributions

**Wei Zhang:** investigation, project administration, writing – review and editing, conceptualization, writing – original draft, data curation. **Linlin Zhou:** data curation, writing – original draft, project administration, visualization. **Yiding Wang:** software, visualization. **Dongbei Guo:** methodology, software, formal analysis. **Hongwei Li:** project administration, writing – review and editing, resources. **Xinyuan Huang:** investigation, visualization, data curation. **Wenqing Bo:** supervision, data curation. **Xinning Zhao:** investigation, validation, formal analysis. **Hanying Zheng:** methodology, validation. **Yingying Zhuang:** project administration, writing – original draft, funding acquisition, conceptualization. **Lili Pan:** supervision, software, methodology. **Xiaoxuan Chen:** methodology, software, supervision. **Hui Shu:** investigation, validation. **Xinyue Wang:** project administration, funding acquisition.

## Funding

This work was supported by the 2024 Xiamen Municipal Guiding Project on Medical and Health Care (No. 3502Z20244ZD1104), the 2025 Xiamen Municipal Guiding Project on Medical and Health Care (No. 3502Z20254ZD1199), and the Fujian Provincial Natural Science Foundation of China: 2025J011505.

## Ethics Statement

All experimental procedures were carried out in accordance with the guidelines of the Institutional Animal Care and Use Committee of the Laboratory Animal Center of Xiamen University and the International Association of Veterinary Editors guidelines for the Care and Use of Laboratory Animals. Protocols for animal use were reviewed and approved by the Animal Ethical and Welfare Committee of the Laboratory Animal Center of Xiamen University (Approval No. XMULAC20240172).

## Conflicts of Interest

The authors declare no conflicts of interest.

## Data Availability

The raw sequencing data from this study have been deposited in the Genome Sequence Archive^34^ in Data Center (https://bigd.big.ac.cn), Beijing Institute of Genomics (BIG), Chinese Academy of Science, under the accession number: CRA030035 and CRA030061.
